# Predictors of malaria rapid diagnostic test positivity in a high burden area of Paletwa Township, Chin State in Western Myanmar

**DOI:** 10.1186/s40249-020-00787-z

**Published:** 2021-01-11

**Authors:** Pyae Linn Aung, Myat Thu Soe, Thit Lwin Oo, Aung Khin, Aung Thi, Yan Zhao, Yaming Cao, Liwang Cui, Myat Phone Kyaw, Daniel M. Parker

**Affiliations:** 1 Myanmar Health Network Organization, Yangon, Myanmar; 2 Myanmar Health Assistant Association, Yangon, Myanmar; 3 Department of Public Health, Ministry of Health and Sports, NayPyiTaw, Myanmar; 4 Department of Immunology, College of Basic Medical Science, China Medical University, Shenyang, 110122 Liaoning China; 5 Division of Infectious Diseases and International Medicine, Department of Internal Medicine, Morsani College of Medicine, University of South Florida, 3720 Spectrum Boulevard, Suite 304, Tampa, FL 33612 USA; 6 Department of Population Health and Disease Prevention, Department of Epidemiology, University of California, Irvine, USA

**Keywords:** Malaria, Trend, Risk, Endemic area, Myanmar

## Abstract

**Background:**

Despite major reductions in malaria burden across Myanmar, clusters of the disease continue to persist in specific subregions. This study aimed to assess the predictors of test positivity among people living in Paletwa Township of Chin State, an area of persistently high malaria burden.

**Methods:**

Four villages with the highest malaria incidence from Paletwa Township were purposively selected. The characteristics of 1045 subjects seeking malaria diagnosis from the four assigned village health volunteers from January to December, 2018 were retrospectively analyzed. Their household conditions and surroundings were also recorded using a checklist. Descriptive statistics and logistic regression models were applied to investigate potential associations between individual and household characteristics and malaria diagnosis.

**Results:**

In 2017, the Paletwa township presented 20.9% positivity and an annual parasite index of 46.9 cases per 1000 people. *Plasmodium falciparum* was the predominant species and accounted for more than 80.0% of all infections. Among 1045 people presenting at a clinic with malaria symptoms, 31.1% were diagnosed with malaria. Predictors for test positivity included living in a hut [adjusted odds ratios (a* OR*): 2.3, 95% confidence intervals (*CI*): 1.2–4.6], owning farm animals (*aOR*: 1.7, 95% *CI:* 1.1–3.6), using non-septic type of toilets (*aOR*: 1.9, 95% *CI:* 1.1–8.4), presenting with fever (*aOR*: 1.9, 95% *CI:* 1.1–3.0), having a malaria episode within the last year (*aOR*: 2.9, 95% *CI:* 1.4–5.8), traveling outside the village in the previous 14 days (*aOR*: 4.5, 95% *CI:* 1.5–13.4), and not using bed nets (a* OR*: 3.4, 95% *CI:* 2.3–5.1). There were no statistically significant differences by age or gender in this present analysis.

**Conclusions:**

The results from this study, including a high proportion of *P. falciparum* infections, little difference in age, sex, or occupation, suggest that malaria is a major burden for these study villages. Targeted health education campaigns should be introduced to strengthen synchronous diagnosis-seeking behaviors, tighten treatment adherence, receiving a diagnosis after traveling to endemic regions, and using bed nets properly. We suggest increased surveillance, early diagnosis, and treatment efforts to control the disease and then to consider the local elimination.
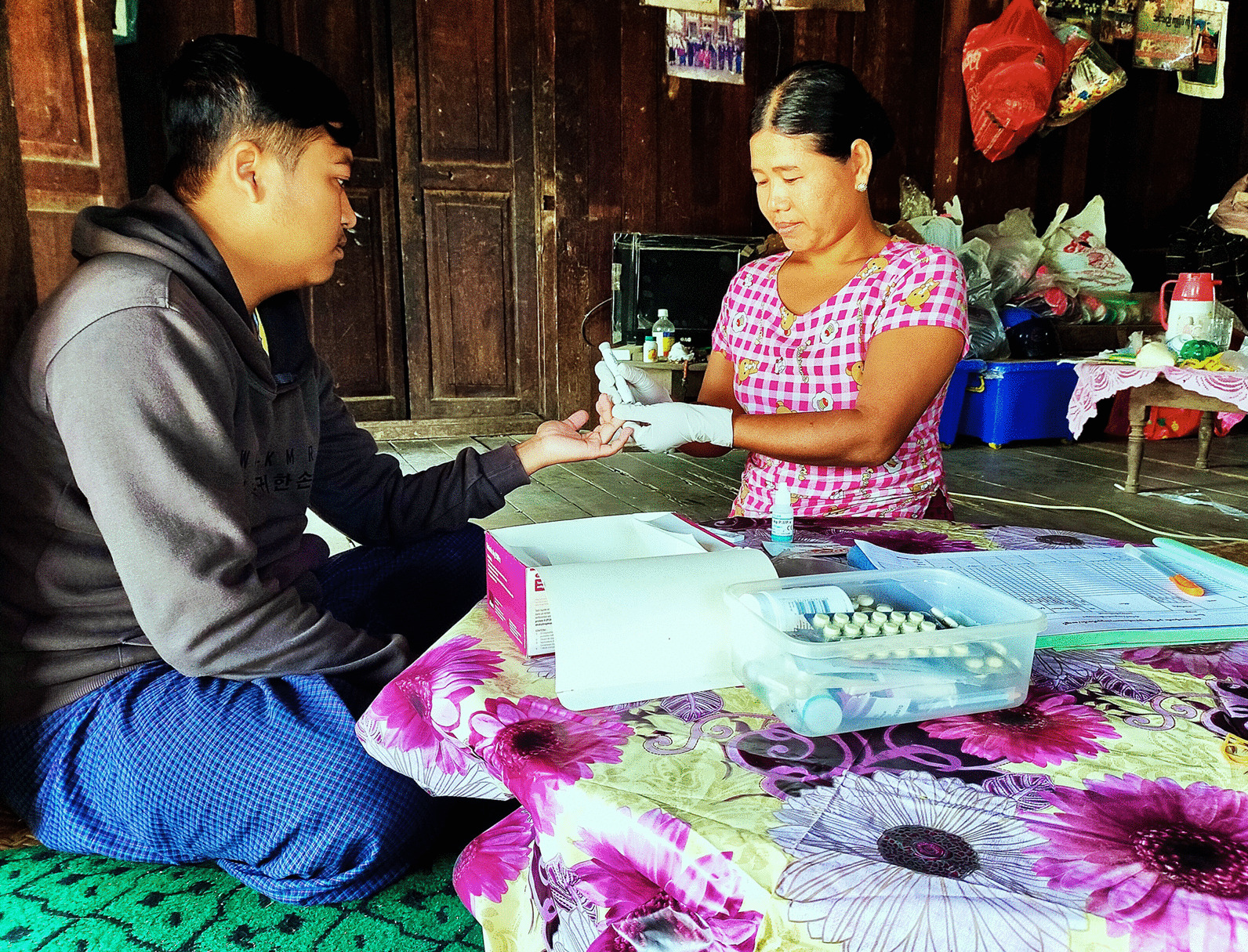

## Background

Although the malaria burden has been declining in Myanmar, morbidity and mortality is still high in specific locations, especially in hard-to-reach rural areas. Since 2011, intensive malaria control activities have rapidly expanded by various partners, including the National Malaria Control Program (NMCP), with the support of different funding sources [1]. As a result, the country is now striving for malaria elimination, intending to be malaria-free by 2030 [2, 3].

Nevertheless, in 2018, more than 74 000 malaria cases were confirmed nationwide [4]. An in-depth analysis revealed that 22.5% of the total cases came from only 12 endemic townships of five states and regions, namely Chin, Kayin, Rakhine, Kachin, and Sagaing [5]. Thus, there is a relatively unchanged disease burden in some specific areas even though there has been an influx of control and elimination-focused activities. An evaluation of the current interventions, and the piloting of newly produced exercises, were employed.

While remoteness and lack of easy access to diagnosis and treatment may partially account for the high malaria burden in some townships, other environmental factors of communities can also predispose them to higher malaria burdens [6, 7]. Communities located in optimal environments for mosquito vectors may have higher baseline risk than places that are unfavorable for mosquito vectors. The presence of environmental attributes that can attract harmful vectors, such as the ownership of farm animals, non-septic toilets, and living in households favorable for *Anopheline* mosquitoes, may contribute to a large burden of disease in particular foci [8–10]. Social and behavioral factors can also be important with regard to risk of infection [11–13].

If such environmental, social, or behavioral characteristics are sufficiently predictive of infection, it may be possible to target high-risk subsets of the population (for example migrant populations, adult males, individuals who have recently travelled, etc.) for public health interventions [14, 15]. Behavioral change communication may be useful for persuading people towards proper prevention practices, especially for use of bed nets or other measures geared toward avoiding exposure to mosquitoes [16]. Conversely, the residents of a village or an area with high transmission may be at equal risk of contracting malaria, regardless of their socio-demographics or behavioral characteristics [17, 18]. In such scenarios, selectively targeted interventions, rather than implementing setting-specific comprehensive malaria control activities, might be ineffective.

This study was aimed at conducting a detailed epidemiological analysis and assessment of socio-economic factors, for the risk of being diagnosed with malaria in an area with a persistently high burden of this disease (Paletwa Township of Chin State, Myanmar). Few detailed studies have focused on this region. Given the national and regional goals of malaria elimination by 2030, understanding drivers of persistently high burdens in specific areas is important for designing and implementing context specific malaria interventions.

## Methods

### Study design and period

Three sources of data were used in this study. First, seven consecutive years (2011–2017) of township-wide data from annual reports of the NMCP were tabulated to examine the overall malaria trend and species composition of Paletwa Township. Second, monthly malaria tests and diagnoses from the Myanmar Health Assistant Association (MHAA) during 2017 and 2018 were used to report the total number of febrile patients, total rapid diagnostic tests (RDTs) confirmed cases, and test positivity. Finally, an individual-level analysis was performed using data collected by village health volunteers (VHVs) in four high burden villages in the setting. All resident villagers presented at the malaria clinics in the four study villages between January and December 2018 were included in this study.

### Study areas

**Fig. 1:**
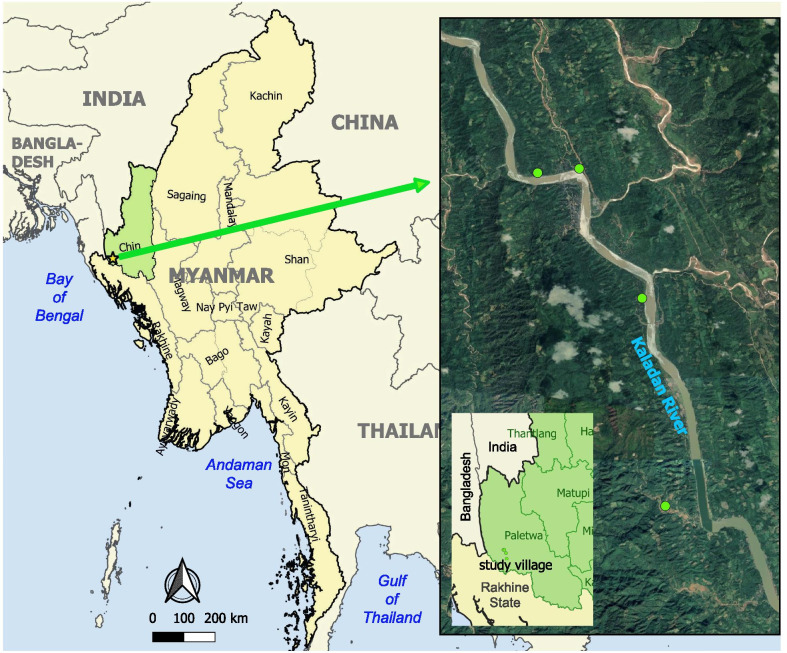
Map indicating study location in Paletwa Township, Chin State, Western Myanmar

The Paletwa Township of Chin State in western Myanmar was purposively selected because it consistently has one of the highest malaria burdens within the country (Fig. 1). The township shares international borders with Bangladesh and India, and also borders Rakhine State. The township has a population of 97 053 according to the 2014 nationwide census, the major ethnic group is the Chin, and most inhabitants are farmers.

For malaria control, the township vector borne disease control (VBDC) team and other partners (such as the Myanmar Medical Association, Medical Action Myanmar, Myanmar Council of Churches, and MHAA) are actively involved in malaria control in the township. Based on available data, it was observed that this township had a consistently high malaria prevalence since 2015, when elimination efforts began in Myanmar. In 2018, the township contributed 64.0% of all malaria cases in Myanmar, with an annual parasite incidence (API) of 15.8/1000 total population at risk (much higher than the targeted pre-elimination goal of API < 1/1000).

The MHAA has been operating in this area since late 2016. To explore the macro-level epidemiology of the study township, malaria data from 100 villages covered by the MHAA were collected. The population coverage was approximately 30.0% of the total population in Paletwa Township.

Next, based on the annual village level data of the MHAA, four villages with the highest malaria test positivity in 2017 were purposively selected to conduct a detailed micro-level epidemiological analysis. All selected high-risk villages were within 7–20 km from the central township area, were located along the riverfronts of Kaladan River, and were located near forests (Fig. 1). The easiest means of transportation from these villages to the nearest large town area is by the river via a boat or shuttle. The total population of the four study villages was 2404 (MeeLatwa village is the largest).

### Data collection and malaria diagnoses

All residents were encouraged to have a blood test soon after showing signs of fever and to strengthen proper preventive measures, especially the use of insecticide-treated nets. For the malaria diagnosis, RDTs detecting *P. falciparum* and *P. vivax* antigens were utilized (SD BIOLINE Malaria Ag Pf/Pv) by VHVs.

The assigned VHV delivered the malaria diagnosis and standard treatment to each suspected patient and recorded the data in a carbonless case report form (CRF). Each CRF addressed the general characteristics of individuals including age in years, sex, presence or absence of fever at the time of diagnosis, RDT result either *P. falciparum* or *P. vivax* or both, and treatment administered. The presence or absence of fever was reported on the basis of the patients’ responses through proper history taking by the VHV. In addition, the VHVs were requested to complete an extra checklist for each febrile patient addressing occupation, education level, travel history of patients, and history concerning malaria attacks, which were not included in the current official CRFs. The occupations of study participants were categorized into the forest or non-forest related types. Goldminers, farmers, and lumberjacks were defined as forest related occupations while government officers, monks, and merchants were represented as non-forest related types of occupation.

Other factors regarding household conditions and the environmental situations were observed and recorded by the study team using a checklist after a person came to the clinic. VHVs visited the patients’ households and each household’s leader was directly asked about the conditions of their households and toilets, and the presence of pigs or cattle during the study year. Households were broadly categorized as hut, manor, or cottage; and toilets were broadly categorized as: non-septic type (pit latrine) or septic type (septic tank or pour-flush latrine) (Additional file 1: Additional figures). In the case that house or toilet conditions changed during the study period, the prevailing conditions were taken into account. Only residents of the study villages were included in this study.

Data assurance and data quality were thoroughly and closely checked by the study team and updated as necessary. The forms were then collected and data were entered in a formatted Microsoft Excel (Excel for Mac, Version 16.16.27, Seattle, USA) spreadsheet using a password protected-computer on a case-by-case basis.

### Data analysis

RDT positivity and API were estimated and plotted for the 100 villages of Paletwa Township by year. Data from the four targeted high-burden villages (January–December 2018) were plotted by month. Summary and descriptive statistics were used to assess the correlates of RDT positivity for febrile patients in the four study villages. Logistic regression (both bi- and multi-variable) was used to investigate potential predictors of RDT positivity among febrile patients in the four study villages. Individuals with repeat episodes were counted only once. All logistic regression results are presented as model adjusted odds ratios (a *OR*) with 95% confidence intervals (*CI*). *P*-values for summary statistics were adjusted using the Bonferroni approach. All analyses were conducted using the Statistical Package for the Social Sciences (IBM SPSS Statistics for Macintosh, Version 23, IBM Corp., Armonk, USA).

### Ethics consideration

The protocol for this study was examined and approved by the institutional review board, Department of Medical Research (Lower Myanmar), and from the University of South Florida, USA.

## Results

### Malaria trends in Paletwa Township from 2011 through 2017

**Fig. 2:**
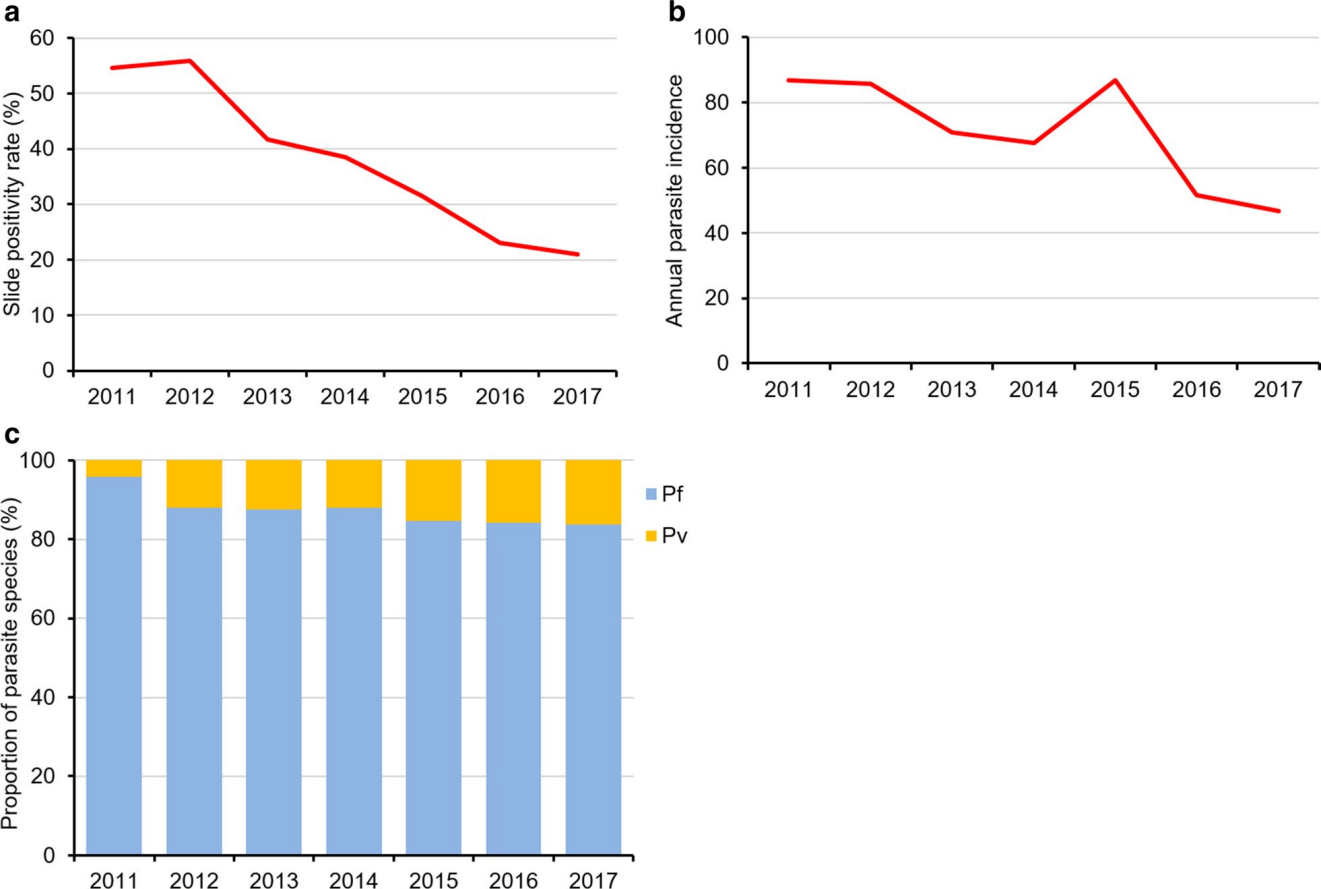
Township level malaria data for Paletwa: **a** Test positivity and **b** annual parasite incidence (2011–2017). **c** *Plasmodium* species proportions (2011–2017). Pf: *Plasmodium falciparum*; Pv: *Plasmodium vivax*

In Paletwa, there was a general reduction of API from 86.8/1000 in 2011 to 46.9/1000 population in 2017 (Fig. 2a). However, the 2017 API in Paletwa was almost 30 times higher than the nationwide API of 1.6. It is noteworthy that the trend was irregular, and the highest API was observed in 2015, when the country started the malaria elimination era and encouraged malaria surveillance system to detect more suspected patients. Similarly, RDT positivity declined progressively each year. While 54.6% of all tests were positive in 2011, 20.9% were positive in 2017 (Fig. 2a). *P. falciparum* was the dominant species reported in Paletwa Township. Among the total 48 144 infections during 2011–2017, almost 88.0% were infected by *P. falciparum*. In each year, *P. falciparum* accounted more than 80.0% of total infections (Fig. 2b). The relative contribution of *P. vivax* to the overall burden of malaria appears to increase over time.

### Malaria trends and seasonality in four high burden study villages

During the study period (January–December 2018), 1045 participants had blood tests by RDTs from four assigned VHVs, and all were eligible to participate in this study. Among them, 325 patients were diagnosed with malaria infections (31.1%). There were five participants with repeat infections during the study period (four with repeated *P. falciparum* episodes and one with a repeat *P. vivax* episode). In the absence of molecular data on the parasite strains, it is unclear whether these were new infections.

RDT positivity in the four study villages declined from 29.1% in 2017 to 24.0% in 2018. Most infections were from *P. falciparum* (73.1%), followed by *P. vivax* (25.0%). Mixed infections accounted for 1.9% of all diagnosed malaria infections. A small increase in the proportion of *P. vivax* cases was observed in 2017 (Table 1).

**Table 1:** Malaria situation in Paletwa Township (2017–2018)

Descriptions	2017	2018	Total
Malaria positivity			
Total febrile cases tested	17 520	16 959	34 479
Confirmed cases	5106	4075	9181
Positivity rate (%)	29.1	24.0	26.6
Malaria parasite [n (%)]			
* Plasmodium falciparum*	3981 (78.0)	2738 (67.2)	6719 (73.1)
* P. vivax*	1020 (20.0)	1272 (31.2)	2292 (25.0)
Mixed infections	105 (2.0)	65 (1.6)	170 (1.9)

The data reflected only for 100 implemented villages by Myanmar Health Assistant Association

**Fig. 3:**
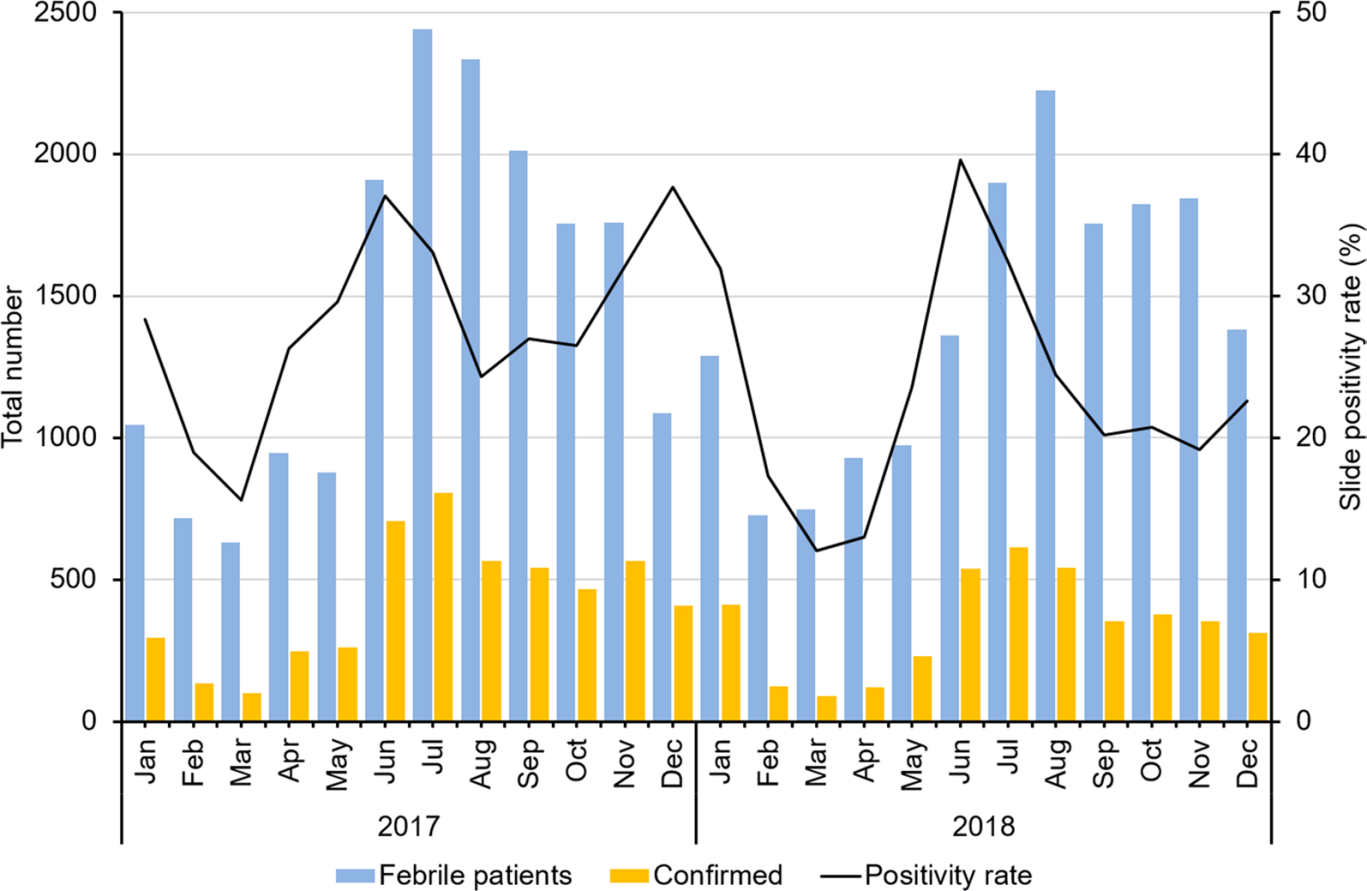
Malaria cases in 2017 and 2018 in the four study villages (including total febrile patients, total confirmed cases, and test positivity)

During the analysis of monthly malaria data for 2017–2018, the disease incidence showed a typical seasonal pattern during the two years. The number of cases was highest in June and lowest in March for both the years. A second peak was also observed in December 2017. The number of diagnoses was the highest at the onset of the rainy season (June–October) and steeply declined after the cold season (November–January) and during the dry season (February–April). Similar fluctuations were observed in RDT positivity (Fig. 3) and total febrile cases.

### Socio-demographic characteristics and risks factors

Most of the febrile patients were below the age of 25 years old (63.2%), and male (53.4%); 53.0% were unemployed (including children, students, and housewives), and 44.4% had forest-related occupations. Few had above primary education (19.0%). The majority of participants lived in huts (93.5%), 94.4% had farm animals (either pig or cattle or both), and 91.2% reported having a non-septic toilet at their home (Table 2 and Additional file 1: Figure S1). A relatively high proportion (32.2%) reported not using bed-nets. Most of the patients (72.3%) presented with fever, few reported having a malaria episode during the last year (6.2%), and 2.6% reported traveling outside of the village within one month before coming to undergo a blood test.

**Table 2:** Descriptive statistics for study participants (*n* = 1045) from four study villages in Paletwa Township, Chin State, Myanmar (January–December 2018)

Description	Frequency (*n*)	Percentage (%)
Malaria infection found by RDT		
Positive cases	325	31.1
* Plasmodium falciparum* (*n* = 325)	251	77.2
* P. vivax* (*n* = 325)	41	12.6
Mixed (*n* = 325)	33	10.2
Negative cases	720	68.9
Population (*n* = 2404) and malaria cases distribution		
LaungKaDuu village (*n* = 674)	83	25.5
MeeLatWa village (*n* = 894)	113	34.8
MonDaungChaungHtel village (*n* = 364)	64	19.7
YokeWa village (*n* = 472)	65	20.0
Age (years)		
< 5	184	17.6
5–14	248	23.7
15–24	229	21.9
≥ 25	384	36.8
Sex		
Male	558	53.4
Female	487	46.6
Occupation		
Unemployed	554	53.0
Forest related workers	464	44.4
Non-forest related workers	27	2.6
Education level		
≤ Primary education	846	81.0
> Primary education	199	19.0
Type of household		
Hut	977	93.5
Others	68	6.5
Having farm animals (cattle/pigs)		
Yes	987	94.4
Type of toilet		
Non-septic	953	91.2
Septic	92	8.8

*RDT* rapid diagnostic test

### Predictors of being diagnosed with malaria

No statistically significant associations were found between malaria diagnosis and age, sex, occupation, and education level of the study subjects. Patients who presented with fever, reported previous malaria episodes, or travelled to outside villages, reported not using bed-nets were more likely to be diagnosed with malaria (*P* < 0.001). Individuals living in huts, especially those with latrine-type toilets, and having livestock were also more likely to be diagnosed with malaria (*P* < 0.001) (Table 3).

**Table 3:** Summary statistics for potential predictors of malaria test positivity among study participants (*n* = 1045) from four study villages in Paletwa Township, Chin State, Myanmar (January–December 2018)

Description	Malaria infection				*P*-value
	Positive		Negative		
	*n*	(%)	*n*	(%)	
Age (years)					
< 5	63	34.2	121	65.8	0.780
5–14	77	31.0	171	69.0	
15–24	69	30.1	160	69.9	
≥ 25	116	30.2	268	69.8	
Sex					
Male	185	33.2	373	66.8	0.273
Female	140	28.7	347	71.3	
Occupation**					
Unemployed	183	33.0	371	67.0	0.519
Forest related workers	133	28.7	331	71.3	
Non-forest related workers	9	33.3	18	66.7	
Education level					
≤ Primary education	249	29.4	597	70.6	0.293
> Primary education	76	38.2	123	61.8	
Type of household					
Hut	282	28.9	695	71.1	< 0.001*
Others	43	63.2	25	36.8	
Having farm animals (cattle/pigs)					
Yes	287	29.1	700	70.9	< 0.001*
No	38	65.5	20	34.5	
Type of toilet					
Non-septic	266	27.9	687	72.1	< 0.001*
Septic	59	64.1	33	35.9	
Having fever					
Yes	261	34.5	495	65.5	0.005*
No	64	22.1	225	77.9	
Having episode of malaria in last year					
Yes	40	61.5	25	38.5	< 0.001*
No	285	29.1	695	70.9	
Traveling to malaria endemic area of outside the village					
Yes	19	70.4	8	29.6	0.001*
No	306	30.1	712	69.9	
Use of bed nets					
Yes	154	21.8	554	78.2	< 0.001*
No	171	50.7	166	49.3	

Degree of freedom for Chi-square test = 1, **Fisher exact test, *P < 0.005, using the Bonferroni correction for multiple tests

Both simple logistic and multi-variable logistic regression analyses revealed similar results (Table 4). The multi-variable logistic regression indicated statistically significant associations between presenting with fever [a *OR* and 95% *CI* of 1.9 (1.1–3.0)], reporting previous malaria attacks [2.9 (1.4–5.8)] and reporting traveling outside their villages in the previous month [4.5 (1.5–13.4)]. In addition, participants who reported not using bed-nets had 3.4 times the odds of being diagnosed with malaria than those who reported using bed nets (95% *CI* 2.3–5.1). Moreover, there were higher odds of malaria infection among some socio-economic characteristics, including: participants living in huts [2.3 (1.2–4.6)], having farm animals [1.7 (1.1–3.6)] and using latrine-type toilets [1.9 (1.1–8.4)].

**Table 4:** Logistic regression models to explore the associations between dependent variables and proneness to have malaria infection (*n* = 1045)

Description	c*OR*	95% *CI*	a*OR*	95% *CI*
Type of household				
Others	1.0		1.0	
Hut	3.2	(1.6–7.4)	2.3	(1.2–4.6)
Having farm animals (cattle/pigs)				
Yes	2.5	(1.4–5.4)	1.7	(1.1–3.6)
No	1.0		1.0	
Type of toilet				
Septic	1.0		1.0	
Non-septic	3.3	(2.1–9.4)	1.9	(1.1–8.4)
Having fever				
Yes	1.9	(1.2–2.9)	1.9	(1.1–3.0)
No	1.0		1.0	
Having episode of malaria in last year				
Yes	3.8	(2.1–7.1)	2.9	(1.4–5.8)
No	1.0		1.0	
Traveling to malaria endemic area of outside the village				
Yes	5.5	(2.1–14.2)	4.5	(1.5–13.4)
No	1.0		1.0	
Use of bed nets				
Yes	1.0		1.0	
No	3.7	(2.5–5.3)	3.4	(2.3–5.1)

*95% CI:* 95% confidence interval, c*OR*: crude odd ratio by simple logistic regression, a*OR*: adjusted odd ratio by multiple logistic regression

## Discussion

The overall malaria positivity in the study reported by MHAA during 2018 was 24.0% (4075 confirmed cases/16 959 total febrile patients). Although Paletwa Township consistently shows a high malaria burden, the situation is heterogeneous, with some villages contributing more to the overall malaria burden than others do. Implementation strategies will benefit from in-depth village-level microstratification of targeted townships whenever possible.

For *P. falciparum* infections, the likelihood of onward transmission decreases when symptomatic individuals quickly receive treatment (as gametocytes take time to develop) [19]. In this study area, therefore, enhanced early diagnosis together with effective treatment compliance, especially by gametocidal and hypnozoiticidal drugs, are needed to interrupt onward locally acquired transmission [20]. The apparent increase in the contribution of *P. vivax* to the overall malaria burden may indicate that diagnosis and treatment efforts have an impact already. Targeted health education campaigns may also improve treatment seeking behaviors among community members [21]. Furthermore, to increase access to early diagnosis and treatment in this remote area, increased implementation of the VHV workforce would be a key activity as suggested in a study from eastern Myanmar [22]. Regular active case detection, either by mobile clinics or diagnosis centers, might also be advantageous for the early detection of disease and to cover the gaps in passive case detection [23, 24]. Interventions such as mass drug administration should only be considered in this setting when a profound surveillance system and proper vector control measures are in place [25].

Among the study participants in the four study villages presented with fever, 34.5% showed positive infections. Fever associated with chill and rigor was seen in more than 90.0% of patients with malaria [26]. In Myanmar, many people seek treatment from unlicensed healers or quacks, potentially leading to onward transmission of the disease and poor health outcomes for infected individuals. Strengthening legislation or introducing methods to improve people’s health understanding and training should be considered to address this problem [27]. Asymptomatic parasite carriers could continue to frustrate control efforts even in the presence of easily accessible diagnosis and treatment [28]. Targeted approaches may be necessary to address asymptomatic reservoirs.

Participants with a history of malaria within the last year had 2.9 times the odds of being diagnosed with malaria than those without a history. One reason for this is that these people may have a forest-related job or live in an environment close to vector breeding sites, making them vulnerable to repeated malaria infections. Also, as Myanmar has many threats to universal health care coverage, some people in this remote area might not be able to use preventive measures such as bed nets or treatment services [29]. Additionally, some recurrent episodes may be the result of relapses or recrudescence (for *P. vivax* infections) [30]. Further, detailed analyses should assess the causes of repeated malaria infections in one person in one single year should also be conducted with follow-up actions taken accordingly.

Traveling outside the village was also a significant predictor of being diagnosed with malaria. While the majority of participants reported using bed-nets, many also reported that when they went outside the village, especially to work, protective measures were neglected [31]. A similar situation was also found at the Thai-Myanmar border, where residual malaria transmission is closely tied to temporary farm huts or shelters during the agricultural season, when people travel to these sites and stay overnight without proper protection [32]. Increasing bed net use and distributing other protective gear (such as insecticide-treated clothes and mosquito repellents) may help address this problem. Diagnosis and treatment efforts that focus on people returning from outside the village, or on people visiting the village, could potentially catch imported infections.

Several studies [18, 33, 34] have shown protective effects of using bed nets. In this study approximately 30% of participants reported not using any nets. Research that focuses on the reasons why people were not using bed nets is warranted. Studies from other regions have illustrated a variety of factors that influence bed net use [35–37]. Procurement policies should account for people’s preferences in addition to other technical aspects of insecticide efficacy and cost-effectiveness.

Household construction and conditions (including the type of toilet and livestock ownership) were associated with malaria infections in this study. Several other studies have shown similar results, in a variety of settings [38–41]. Further work on the ecology of malaria vectors in this region may help to explain this association better. One vector study [42] in Myanmar reported that in the study township (Paletwa), *Anopheles minimus* served as the major vector. Adults preferred to rest in houses, were anthropophilic and fed beginning at dusk with a peak around midnight (both outdoors and indoors) and a gradual decrease in feeding activity toward the dawn (6:00 am). *An. maculatus*, *An. sinensis*, *An. philippinensis*, *and An. annularis* were suggested to be secondary vectors, feeding both humans and animals. Interventions that consider household design and construction may therefore be warranted.

Several studies from the Greater Mekong Subregion have shown that *P. falciparum* infections are frequently acquired among adult males [43, 44]. In the current study, however, the respondents’ age was inconsequential to the risk of being diagnosed with malaria. This finding corresponds to one from areas with a high malaria burden in eastern Myanmar [45]. Once active foci are eliminated through extensive malaria control measures (including expansion of access to diagnosis and treatment facility), *P. falciparum* transmission persists in remote areas away from the village—becoming an occupational hazard. Subsequently, *P. falciparum* cases are mostly observed among adult males who were probably infected from somewhere else outside the village [46].

This study has some limitations. Some villagers may have been diagnosed and treated elsewhere and would not have been detected in this study. The presence of malaria infection was confirmed only by RDTs as the study area had no access to microscopy examination. There may be asymptomatic infections in the community, which are unlikely to be detected by the VHV. In order to understand the total malaria burden in this setting it would be valuable to conduct a prevalence survey with highly sensitive screening tests. Febrile illnesses that were not diagnosed as malaria did not receive final diagnoses, as the VHVs are limited in their diagnostic capabilities. Further work that includes more detailed and longitudinal assessments of the predictors of malaria infections in this region are warranted.

## Conclusions

The results of this study suggested potential risk factors to be aware of in preventing malaria infection among people living in this high burden area. People living in huts, with non-septic toilets, possessing farm animals, presenting with fever, having a malaria episode in the last year, traveling to any outside village in the last 14 days and not using bed nets were more likely to have a positive malaria test by RDT. In addition to current exclusive malaria control activities, modifying these factors may also reduce caseloads. To improve access to malaria diagnosis and treatment, services should also be encouraged by expanding the VHV workforce. Furthermore, a large proportion of febrile illnesses were not diagnosed as malaria. The causative agent behind these illnesses normally remains unknown. As these illnesses are likely to remain while the malaria burden decreases, it is important to address these non-malarial febrile illnesses. Targeted health education campaigns should be introduced to strengthen synchronous diagnosis-seeking behaviors, tighten treatment adherence, receiving a diagnosis after traveling to endemic regions, and using bed nets properly. On the other hand, the economic index of a community should be improved by collaborating with local government agencies, non-state actors, and other community development organizations. Finally, special attention should be paid to this and other high burden areas so that they do not frustrate national elimination efforts.

## Supplementary Information

**Additional file 1:** Additional figures.

## Data Availability

All data analyzed for this study are included within the article.
