# Supplementary material for: Predictors of malaria rapid diagnostic test positivity in a high burden area of Paletwa Township, Chin State in Western Myanmar
Source: Infect Dis Poverty. 2021 Jan 11;10:6. doi: 10.1186/s40249-020-00787-z (PMC7802189; doi:10.1186/s40249-020-00787-z)

**ADDITIONAL FILE**

**Description of village health volunteers (VHV)**

The MHAA is a local nongovernmental organization (NGO) that implements malaria control activities, with funding support from various sources. A sub-office was opened by the MHAA and was occupied by three assigned health assistants and five field supervisors. The staff is paid a monthly salary directly from the central office of Yangon, Myanmar and is monitored by higher-level staff. A VHV was assigned to each village by the MHAA without overlapping other partners, including the respective township’s VBDC team. In addition to the VHVs’ activities, routine health education sessions were in place in each village, organized by the MHAA’s field staff using pamphlets and posters.

The MHAA trained VHVs during a 5-day recruitment training session and 3 to 4 days of yearly refresher training and organized routine supportive supervision. With guidance from the NMCP, the volunteers were remunerated with a standardized incentive scheme of 50,000 MMK (35 USD) per quarter, and other occasional in-kind distributions. Moreover, full supplies for malaria diagnosis and treatment were ensured and refilled when necessary to avoid potential stock-outs. The training-aid materials, checklists, case report forms (CRFs) , and other formats were adopted and reproduced from the NMCP’s standard materials and guidelines.

**ADDITIONAL FIGURES**

**Additional Figure S1: Example of simple latrine-type toilet**


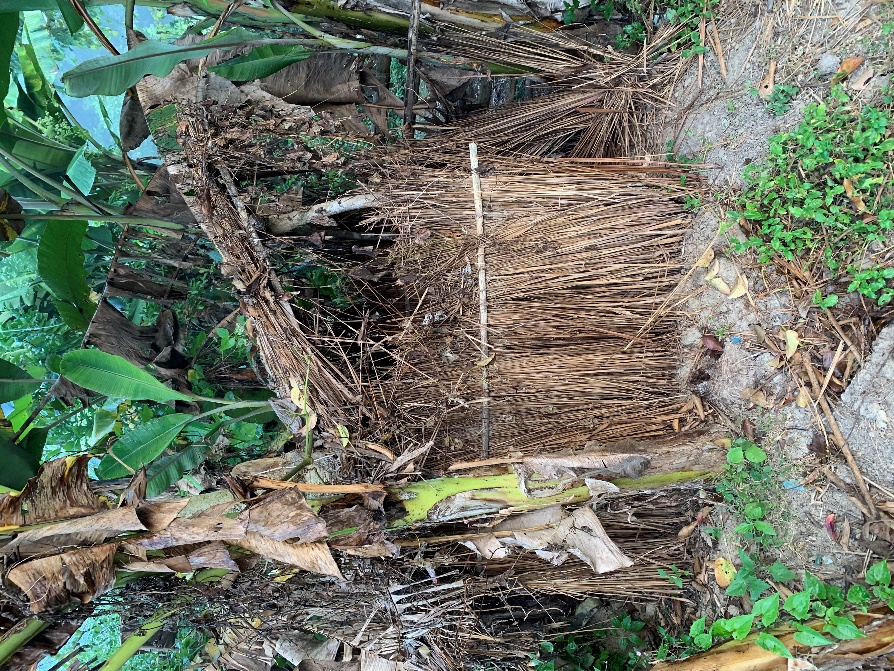


**Additional Figure S2: Example of a hut**


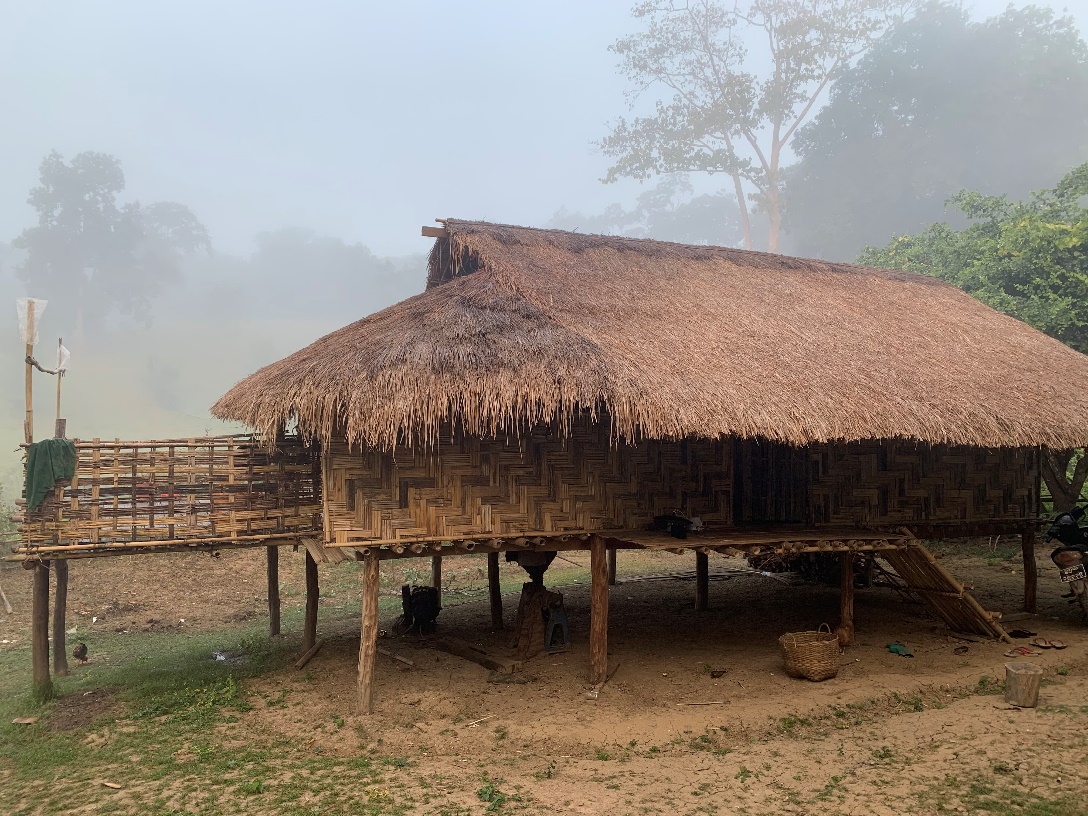


**Additional Figure S3A: Example of a house that is not considered as hut**


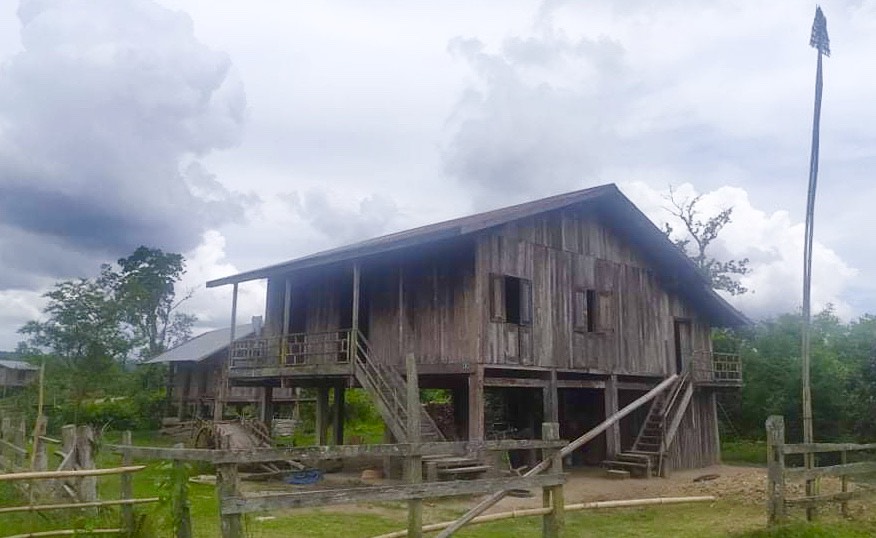


**Additional Figure S3B: Example of a house that is not considered as hut**


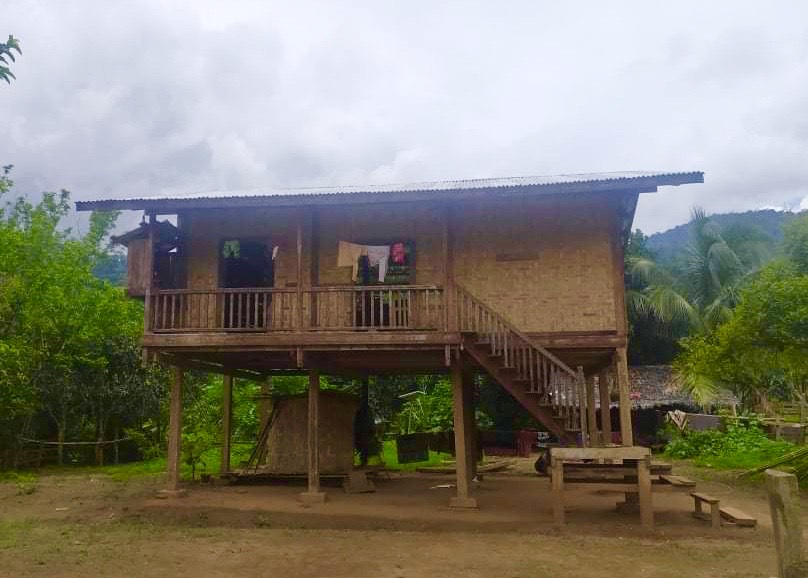

Supplement: Supplementary file 1 — Additional file 1: Additional figures. [file 40249_2020_787_MOESM1_ESM.docx]
